# Propagation velocity profile in a cross-section of a cardiac muscle bundle from PSpice simulation

**DOI:** 10.1186/1742-4682-3-29

**Published:** 2006-08-15

**Authors:** Nicholas Sperelakis, Lakshminarayanan Ramasamy

**Affiliations:** 1 Dept. of Molecular & Cellular Physiology, University of Cincinnati College of Medicine, Cincinnati, OH 45267-0576, USA; 2 Dept. of Electrical Computer Engineering and Computer Science, University of Cincinnati College of Engineering, Cincinnati, OH 45219, USA

## Abstract

**Background:**

The effect of depth on propagation velocity within a bundle of cardiac muscle fibers is likely to be an important factor in the genesis of some heart arrhythmias.

**Model and methods:**

The velocity profile of simulated action potentials propagated down a bundle of parallel cardiac muscle fibers was examined in a cross-section of the bundle using a PSpice model. The model (20 × 10) consisted of 20 chains in parallel, each chain being 10 cells in length. All 20 chains were stimulated simultaneously at the left end of the bundle using rectangular current pulses (0.25 nA, 0.25 ms duration) applied intracellularly. The simulated bundle was symmetrical at the top and bottom (including two grounds), and voltage markers were placed intracellularly only in cells 1, 5 and 10 of each chain to limit the total number of traces to 60. All electrical parameters were standard values; the variables were (1) the number of longitudinal gap-junction (G-j) channels (0, 1, 10, 100), (2) the longitudinal resistance between the parallel chains (R_ol2_) (reflecting the closeness of the packing of the chains), and (3) the bundle termination resistance at the two ends of the bundle (R_BT_). The standard values for R_ol2 _and R_BT _were 200 KΩ.

**Results:**

The velocity profile was bell-shaped when there was 0 or only 1 gj-channel. With standard R_ol2 _and R_BT _values, the velocity at the surface of the bundle (θ_1 _and θ_20_) was more than double (2.15 ×) that at the core of the bundle (θ_10_, θ_11_). This surface:core ratio of velocities was dependent on the values of R_ol2 _and R_BT_. When R_ol2 _was lowered 10-fold, θ_1 _increased slightly and θ_2_decreased slightly. When there were 100 gj-channels, the velocity profile was flat, i.e. the velocity at the core was about the same as that at the surface. Both velocities were more than 10-fold higher than in the absence of gj-channels. Varying R_ol2 _and R_BT _had almost no effect. When there were 10 gj-channels, the cross-sectional velocity profile was bullet-shaped, but with a low surface/core ratio, with standard R_ol2 _and R_BT _values.

**Conclusion:**

When there were no or few gj-channels (0 or 1), the profile was bell-shaped with the core velocity less than half that at the surface. In contrast, when there were many gj-channels (100), the profile was flat. Therefore, when some gj-channels close under pathophysiological conditions, this marked velocity profile could contribute to the genesis of arrhythmias.

## Background

It is predicted from cable theory that velocity of propagation along a fiber is a function of the external resistance of the fluid bathing the fiber: the higher the resistance the slower the velocity [1]. When parallel fibers are packed within a small-diameter bundle, the outside resistance of fibers near the core should be greater than that of fibers at the surface. Therefore, it is predicted that, by recording electrically at different depths within a myocardial bundle, the propagation velocity of the deeper fibers should be slower than that of the surface fibers. This phenomenon would occur presumably because of the high longitudinal resistance of the interstitial space (or R_ol2_), which reflects the tightness of packing of the parallel fibers within the bundle. Consistent with this, measurements of tissue resistivity in the longitudinal direction vs. transverse (radial) direction showed a marked asymmetry, the resistivity being much higher in the transverse direction [2].

Wang et al. [3] carried out a simulation study of a tightly-packed cardiac muscle bundle and found a large interstitial potential; the central (core) fiber exhibited a much slower propagation velocity than the surface fiber when there was no transverse coupling (i.e. no gj-channels) between the fibers. When there was transverse coupling, the central fiber and surface fiber had the same velocity. Other simulation studies of propagation in a cardiac muscle bundle were carried out by Henriquez and Plonsey [4-6].

Such slowing of the propagation velocity within the depths of cardiac bundles may be an important factor in the genesis of certain arrhythmias under some pathophysiological conditions, such as ischemia. Therefore, the present experiments were carried out on a cardiac muscle bundle model, using PSpice to analyze the propagation of simulated cardiac action potentials (APs) at different depths within the bundle. It was found that when there were no or few gj-channels, the velocity profile was bell-shaped, with the velocity at the core of the bundle more than 2-fold slower than at the surface. Since the profile was flat when there were many gj-channels, any change in number of gj-channels caused by pathophysiological conditions could contribute to certain arrhythmias.

## Methods

**Figure 1:**
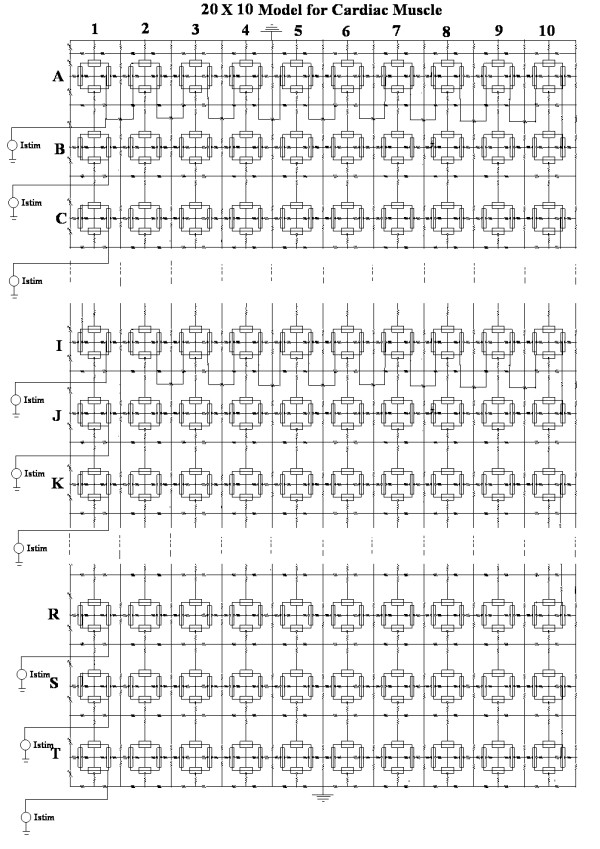
Electrical circuit of the 20 × 10 model (20 parallel chains of 10 cells each) of a cardiac muscle bundle used for determining the cross-sectional profile of longitudinal propagation velocities. The simulated bundle was symmetrical at the top and bottom, including values of R_ol _and R_or _and the presence of two grounds. All 20 chains were stimulated intracellularly and simultaneously by the 20 stimulators at the left end of the bundle using rectangular current pulses (0.25 nA, 0.25 ms). To prevent cluttering of the diagram, the R_gj _resistors are shown only for chain A. Voltage markers were placed intracellularly only in cells 1, 5 and 10 of each chain so as to limit the total number of traces to 60. The variables were: (a) the number of gj-channels placed across the longitudinal cell-to-cell junctions in each chain (R_gj_), (b) the longitudinal resistance of the interstitial fluid between the parallel chains (R_ol2_), and (c) the bundle termination resistance at the ends of the bundle (R_BT_).

The circuit details, including that of the basic units representing patches of excitable membrane, have been given in our previous papers [7-10]. For the present experiments, the model of cardiac muscle consisted of 20 chains in parallel, each chain being 10 cells in length (20 × 10 model) (Fig. 1). The model was intended to represent a cross-sectional plane through a segment of the central core of a cardiac muscle bundle of small diameter. To this end, the top and bottom of the model were made symmetrical, including identical R_ol _and R_or _values and two grounds to reflect the upper and lower surfaces of the bundle (Fig. 1). Twenty identical electrical stimulators were placed on the left end of the model so that all 20 chains could be stimulated simultaneously. The rectangular current pulses were all identical, i.e. 0.25 nA in amplitude and 0.25 ms in duration, and stimulation was applied intracellularly. Voltage recordings (markers placed intracellularly) were made only from cells 1, 5 and 10 of each chain in order to limit the total number of traces to 60 (20 chains × 3 markers/chain).

All electrical parameters were the standard values; the variables were as follows. One variable was the number of gap-junction (gj) channels inserted at the cell junctions in each chain. This number was varied from 0 to 1, 10 and 100, with each gj-channel assumed to be 100 pS. Another variable was the value of the longitudinal resistance of the interstitial fluid space between the parallel chains (R_ol2_). The R_ol2 _value reflects the closeness of packing of the chains: the higher the value, the tighter the packing. The standard value of R_ol2 _was 200 KΩ. The third variable was the bundle termination resistance (R_BT_) at the two ends of the bundle. The standard value for R_BT _was 200 KΩ.

The longitudinal propagation velocity (θ) was calculated from the measured total propagation time (TPT), assuming a cell length of 150 μm, from the following equation:

Θ=9 junc×15.0×10−3cm/juncTPT(ms)
 MathType@MTEF@5@5@+=feaafiart1ev1aaatCvAUfKttLearuWrP9MDH5MBPbIqV92AaeXatLxBI9gBaebbnrfifHhDYfgasaacH8akY=wiFfYdH8Gipec8Eeeu0xXdbba9frFj0=OqFfea0dXdd9vqai=hGuQ8kuc9pgc9s8qqaq=dirpe0xb9q8qiLsFr0=vr0=vr0dc8meaabaqaciaacaGaaeqabaqabeGadaaakeaacqqHyoqucqGH9aqpdaWcaaqaaiabiMda5iabbccaGiabbQgaQjabbwha1jabb6gaUjabbogaJjabgEna0kabigdaXiabiwda1iabc6caUiabicdaWiabgEna0kabigdaXiabicdaWmaaCaaaleqabaGaeyOeI0IaeG4mamdaaOGaee4yamMaeeyBa0Maei4la8IaeeOAaOMaeeyDauNaeeOBa4Maee4yamgabaGaeeivaqLaeeiuaaLaeeivaqLaeiikaGIaeeyBa0Maee4CamNaeiykaKcaaaaa@536F@

Therefore, the velocity measured was the average velocity, not the instantaneous velocity.

## Results

**Figure 2:**
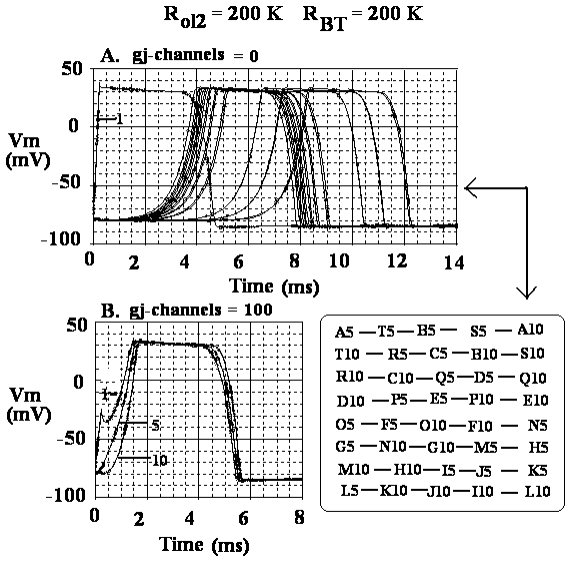
Action potential (AP) traces recorded intracellularly in cells 1, 5, and 10 of each of the 20 parallel chains of the cardiac bundle. The order of firing for panel A (cells 5 and 10 of each chain) is given by the inset table at the lower right of the figure. A Zero gj-channels. The first visible trace consists of the superimposition of the 20 AP traces recorded from cell #1 of all 20 chains. B: 100 gj-channels. Each of the three traces visible (cells # 1, 5 and 10, respectively) consists of the superimposition of the 20 APs of the 20 parallel chains.

**Figure 3:**
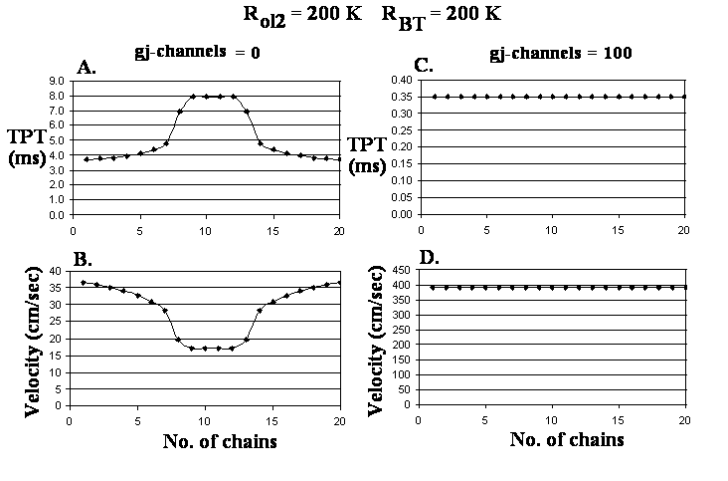
Graphs of the measured total propagation time (TPT) (A, C) and calculated longitudinal propagation velocity (B, D) through the cross-section of the cardiac bundle. A-B: Zero gj-channels. The cross-sectional profile through the core of the bundle is bell-shaped. The velocity at the bundle surface is about double that at the bundle core.

The 60 traces recorded from cells #1, 5 and 10 of the 20 parallel chains are shown in Figure 2A for zero gj-channels. The first trace is the superimposition of the 20 APs from cell #1 of each chain. The remaining traces are identified in the table inserted into this panel (A). The total propagation times (TPT) for the impulses to reach cell #10 of each chain are plotted in Figure 3A. Note that this curve is bell-shaped, and TPT varies from about 3.7 ms at the two surfaces of the bundle to almost 8.0 ms at the core. From these TPT data, the velocities for longitudinal propagation were calculated and plotted in Figure 3B. Again, note that the curve is bell-shaped, the velocity being about 36.5 cm/s at the surface and 17.0 cm/s at the core. Thus, propagation velocity was more than 2-fold faster (2.15) at the surface than at the core. These data are summarized in Table 1, category A.

**Table 1:** Summary of experiments to determine the cross-sectional profile of impulse propagation in a bundle of cardiac fibers (20 × 10 model).

	**No. of Gj-channels**	**R**_ol2 _**(KΩ)**	**R**_BT _**(KΩ)**	**Θ**_1 _**(cm/sec)**	**Θ**_10 _**(cm/sec)**	**Θ**_1 _**/Θ**_10 _**Ratio**	**Shape**
**A**	0	200	200	36.6	17.0	2.15	Bell-shaped
**B**	1	200	200	36.8	19.7	1.86	Bell-shaped
**C**	10	200	200	46.6	44.6	1.04	Bullet-shaped
**D**	100	200	200	397	397	1.00	Flat
**E**	0	2000	200	32.9	29.0 ^#^28.1	1.13 1.17	Accent bullet-shaped dimple
**F**	10	2000	200	46.6	44.6	1.04	Bullet-shaped
**G**	100	2000	200	397	397	1.00	Flat
**H**	0	2000	2000	62.2	32.9	1.89	Bell narrower and **Θ **approx doubled
**I**	100	2000	2000	386	397	0.97	Flat
**J**	0	20	200	38.1	14.4	2.65	Bell-shaped
**K**	0	20	20	13.0	14.4	0.90	Inverse
**L**	10	20	200	46.7	43.8	1.07	bell-shaped, but low ratio
**M**	100	20	200	397	397	1.00	Flat
**N**	0	50	200	37.4	15.4	2.43	Bell-shaped
**O**	0	800	200	35.1	22.7	1.55	Bell-shaped

The gj-channels were inserted at the cell junctions of each chain.

Θ_1 _---- velocity at edge of bundle.

Θ_10 _---- velocity at middle of bundle.

20 × 10 model ---- 20 parallel chains of 10 cells each.

All 20 cells at left end of bundle were stimulated simultaneously using rectangular current pulses of 0.25 nA and 0.25 ms duration.

Bundle was symmetrical at the top and the bottom (including two grounds).

All parameters were set at standard values, including R_jc _of 25 MΩ (50 MΩ ÷ 2).

Standard value for R_ol2 _was 200 KΩ, and for R_BT _was 200 KΩ.

Voltage markers were placed only on cells 1, 5 and 10 of each chain (i.e., total of 60 traces).

# This second value is for θ_6 _(also θ_15_)_, _because this is where maximum slowing occurred (because of the dimple).

Figure 2B shows the 60 traces recorded when 100 gj-channels were inserted longitudinally at the cell junctions in each chain. Three traces can be seen, the first being the superposition of 20 traces from cell #1 of each chain, and the second and third being the superposition of 20 traces each from cells #5 and #10 of each chain, respectively. These results are plotted in Figure 3C for TPT and 3D for propagation velocity. The curves are flat, TPT being about 0.35 ms and velocity being about 400 cm/s for both the surface and core fibers. These data are summarized in Table 1, category D.

**Figure 4:**
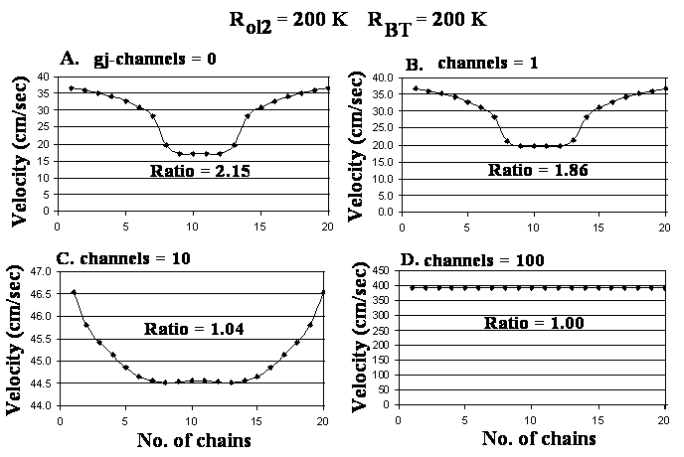
Graphs of the cross-sectional profile through a small-diameter cardiac bundle of the propagation velocities for different numbers of gj-channels: 0 (A), 1 (B), 10 (C), and 100 (D). All other parameters were standard, including R_ol2 _(200 KΩ) and R_BT _(200 KΩ). Panel A is the same as panel B of Fig. 3, but it is included again here to facilitate comparison. The profile is bell-shaped (inverted) in A and B, bullet-shaped in C, and flat in D. In C, the velocity at the bundle surface is only slightly faster than that at the core.

Figure 4 shows the propagation velocity profiles that were obtained when the number of gj-channels was varied from 0 (A) to 1 (B), 10 (C) and 100 (D), while R_ol2 _and R_BT _had the standard values (200 KΩ for both). These data are summarized in Table 1 (categories A-D). Note that the bell-shaped profile (A, B) changed to bullet-shaped (C) and to flat (D). Also note that the ratio of velocities (surface to core) was low compared to that in panels A and B. This indicates that adding gj-channels (10 or 100) flattened the profile.

**Figure 5:**
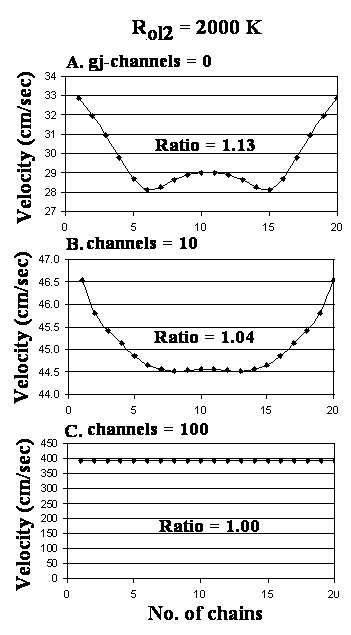
Graphs of the cross-sectional profile through a small-diameter cardiac bundle of the propagation velocities for different numbers of gj-channels: 0 (A), 10 (B) and 100 (C). The longitudinal resistance of the interstitial fluid between the 20 parallel chains (R_ol2_) was elevated 10-fold to 2000 KΩ (from the standard 200 KΩ). The main difference in the profiles, compared to when R_ol2 _was 200 KΩ (Fig. 4), is the widening and dimpling of the bell in panel A.

Figure 5 gives the velocity profiles obtained when the number of gj-channels was varied from 0 (A) to 10 (B) and 100 (C), with R_ol2 _increased 10-fold (to 2000 KΩ). These data are summarized in Table 1 (categories E, F, G). Note that raising R_ol2 _greatly diminished the surface to core ratio of velocities and the profiles became bullet-shaped in A and B. Also note that there was a "dimpling" of the bullet shape at the core region.

**Figure 6:**
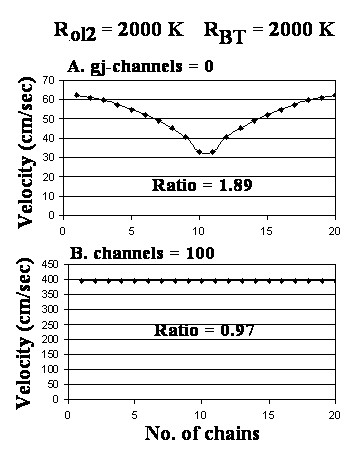
Graphs of the bundle cross-sectional velocity profile when both R_ol2 _and the bundle termination resistance (R_BT_) were increased 10-fold to 2000 KΩ. A: 0 gj-channels. B: 100 gj-channels. When there were 0 channels, the bell was narrowed, and with 100 channels there was very little effect (compare with Fig. 4D).

Figure 6 shows the effect of elevating R_BT _10-fold (to 2000 KΩ), in addition to the 10-fold elevation of R_ol2_, for 0 (A) and 100 (B) gj-channels. When there were no gj-channels (A), the bell narrowed at the core region and the velocity at both the surface and the core increased greatly (about doubled). The changes in resistance had almost no effect when there were 100 gj-channels (B). These data are summarized in Table 1 (categories H and I).

**Figure 7:**
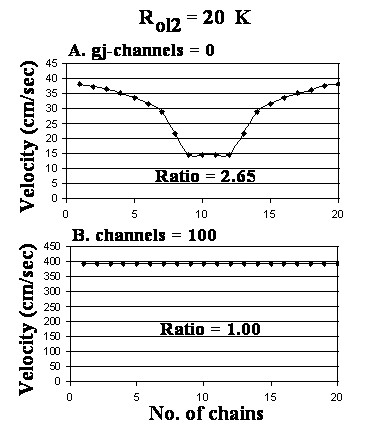
Graphs of the bundle cross-sectional velocity profile when R_ol2 _was lowered 10-fold to 20 KΩ. A: 0 gj-channels. B: 100 gj-channels. When there were 0 channels, the shape was similar to that when R_ol2 _was the standard 200 KΩ (see Fig. 4A). With 100 channels, there was almost no effect (compare with Fig. 4D).

Figure 7 shows that lowering R_ol2 _10-fold (to 20 KΩ) for 0 (A) and 100 (B) gj-channels had almost no effect. These data are summarized in Table 1 (categories J and M).

## Discussion

The present PSpice analysis of the cross-sectional profile of longitudinal propagation velocities of simulated APs through a small-diameter bundle of cardiac muscle fibers indicates that velocity is lower in the depths of the bundle than at the surface. This difference was apparent when there were 0, 1 or 10 gj-channels at the cell junctions. The cross-sectional profile was bell-shaped when there were 0 or 1 gj-channels and bullet-shaped when there were 100 channels. The ratio of the velocity at the bundle surface to that at the bundle core was over 2.0 when there were 0 or 1 gj-channels (Table 1 A, B). This ratio was greatly reduced when there were 10 channels (Table 1). With 100 channels, the ratio was reduced to 1.00 and the cross-sectional profile was flat (Table 1 C).

Increasing the value of the longitudinal resistance of the interspace between the parallel chains (R_ol2_) 10-fold to 2000 KΩ greatly accelerated propagation at the core, and so reduced the ratio, when there were 0 gj-channels (Table 1E). When there were 10 or 100 channels, the elevation of R_ol2 _had very little effect (Table 1 F, G).

Increasing both the bundle termination resistance (R_BT_) and R_ol2 _10-fold (each to 2000 KΩ) greatly accelerated (almost doubled) the velocity of propagation at both the bundle surface and the core, but the surface/core ratio remained high when there were 0 gj-channels (Table 1 H). When there were 100 channels, there was almost no effect (Table 1 I).

Lowering R_ol2 _by 10-fold (to 20 KΩ) had only a small effect at 0 gj-channels (Table 1 J) and at 10 or 100 channels (Table 1 L, M). When R_BT _was also lowered 10-fold (to 20 KΩ), the propagation velocity was greatly reduced at the surface at 0 gj-channels but was almost unaffected at the core, and the surface/core ratio was reduced to less than 1.00 (Table 1 K).

Lowering R_ol2 _4-fold (to 50 KΩ) had almost no effect (Table 1 N). Raising R_ol2 _4-fold (to 800 KΩ) had an intermediate effect (Table 1 O), i.e. it increased the propagation velocity in the core and thereby reduced the surface/core ratio.

Thus, when cell-to-cell transmission is by the electric field (EF) mechanism (0 or 1 gj-channel), the surface/core ratio is high (about 2.0). This means that propagation velocity at the core of the bundle is about half that at the surface. In contrast, when cell-to-cell transmission is by local-circuit currents through gj-channels, the surface/core ratio is about 1.0 and the cross-sectional profile is flat. Hence, propagation velocity is uniform at all depths of the bundle. Since non-uniform velocities could contribute to re-entrant types of arrhythmias, any decrease in the number of functional gj-channels under pathophysiological conditions (such as transient ischemia) might give rise to arrhythmias.

As expected, the velocity of propagation increases as more and more gj-channels are inserted (compare A, B, C and D of Table 1). The velocity increased from 36.6 cm/s (0 channels) to 36.8 cm/s (1 channel), 46.6 cm/s (10 channels) and 397 cm/s (100 channels). The last of these values is well above that measured physiologically. In adult canine atria, the longitudinal conduction velocity varies from about 85 to 105 cm/s (depending on cycle length), and in infant atria the range is about 35 to 50 cm/s; the transverse velocity varied from 11 to 18 cm/s for adults and 8 to 14 cm/s for infants [11].

Note that when R_ol2 _was increased 10-fold, the core velocity increased greatly (from 17.0 cm/s to 29.0 cm/s) when there were zero gj-channels (Table 1 E vs. 1 A). This effect caused the surface/core ratio to drop from 2.15 to 1.13. Hence, when cell-to-cell transmission of excitation is by the EF mechanism [12], raising R_ol2 _increases the velocity, consistent with our previous finding [13]. Since the surface fibers are exposed to R_ol_and R_or_, not to R_ol2_, the velocity at the surface does not change. Consistent with this interpretation, when both R_BT _and R_ol2_, were raised 10-fold, the velocity at the surface greatly increased as well, bringing the surface/core ratio back close to 2.0 (Table 1H). Lowering R_ol2 _10-fold had almost no effect (Table 1 J, L). In contrast, lowering R_BT _10-fold when there were zero gj-channels produced a large decrease in velocity at the surface, thus decreasing the surface/core ratio to 0.9 (Table 1 K).

We cannot explain the finding of a bell-shaped profile (for 0 or 1 gj-channel) (Fig 3 A, B; Fig 4 A, B). We expected a bullet-shaped profile under the standard parameters. However, when R_ol2 _was increased 10-fold, the profile changed from bell-shaped to bullet-shaped (with a dimple) (Fig. 5 A). The profile was bullet-shaped under standard parameters when there were 10 gj-channels (Fig. 4C), but the surface/core velocity ratio was low. This profile remained bullet-shaped even when R_ol2 _was raised 10-fold (Fig 5 B).

The present results using PSpice analysis are in very good agreement with those reported by Wang et al. [3], who used a computer model with programs written in C language. Their studies showed that, when there was no transverse coupling between the fibers (chains) in the cardiac muscle bundle, the velocity of propagation in the core fiber was much lower than that in the surface fiber. (In their model, the myocardial cells were very long, so many longitudinally-oriented gj-channels were, in effect, present.) When the distance between the parallel fibers was 100 Å or less, there was a large interstitial potential (equivalent to our EF mechanism), which increased in magnitude as the distance was reduced (equivalent to increasing R_ol2 _in the present study). When there was strong transverse coupling between the parallel chains, the propagation velocity in the core chain was the same as that in the surface chain, as found in the present study.

In summary, the present study demonstrates that longitudinal propagation velocity in a simulated small-diameter bundle of cardiac muscle is markedly lower in the depths and core of the bundle than at the surface. However, such slower propagation occurs only when there are no or few gj-channels (0, 1 or 10). When there were many gj-channels, the velocity profile was flat and the surface/core ratio of velocities was 1.0. Therefore, under pathophysiological conditions that can render some gj-channels non-functional, the observed phenomenon can lead to reentrant arrhythmias. The finding by Poelzing et al. [14,15] that there is heterogeneous expression of connexin43 across the ventricular wall of canine heart, and that this may produce arrhythmias in heart failure, provides some evidence that alterations in the number of functioning gj-channels can have serious consequences.
